# Moving beyond the Slit-Lamp Gonioscopy: Challenges and Future Opportunities

**DOI:** 10.3390/diagnostics11122279

**Published:** 2021-12-06

**Authors:** Carlo Alberto Cutolo, Chiara Bonzano, Riccardo Scotto, Michele Iester, Alessandro Bagnis, Chiara Pizzorno, Carlo Catti, Carlo Enrico Traverso

**Affiliations:** 1 Clinica Oculistica, Department of Neuroscience, Rehabilitation, Ophthalmology, Genetics, Maternal and Child Health, University of Genoa, 16132 Genoa, Italy; oculistabonzano@gmail.com (C.B.); riccardoscotto@hotmail.com (R.S.); iester@unige.it (M.I.); alebagnis@libero.it (A.B.); chiara.pizzorno992@gmail.com (C.P.); Carlo.III@hotmail.it (C.C.); cet@mclink.it (C.E.T.); 2 IRCCS Ospedale Policlinico San Martino, 16132 Genoa, Italy

**Keywords:** gonioscopy, anterior chamber angle, iridocorneal angle

## Abstract

After almost a century from its introduction in clinical practice, slit-lamp gonioscopy is still considered the reference standard for evaluating the anterior chamber angle (ACA). Gonioscopy is essential for diagnosing angle closure disease, and ACA features are included in glaucoma’s diagnostics and treatments algorithms. However, shortcomings of slit-lamp gonioscopy include a steep learning curve, lack of agreement between examiners and poor documentation. Thanks to advances in miniaturization and computing, new instruments for digital gonioscopy have been developed and marketed. This narrative review focuses on the Gonioscope GS-1, which permits semi-automated circumferential documentation of the ACA in real-colour photographs. Advantages and disadvantages of GS-1 compared with slit-lamp gonioscopy and other ACA imaging technologies such as optical coherence tomography are discussed. Finally, potential opportunities offered by this device for telemedicine, virtual clinics, and automatic classification with deep learning are presented.

## 1. Introduction

Slit-lamp gonioscopy is an essential part of adult eye examination to detect angle-closure or define secondary causes of intraocular pressure (IOP) elevation [1]. Moreover, many treatments targeted at the anterior segment of the eye are delivered through gonioscopy lenses and not performing gonioscopy in patients suspected of having glaucoma may cause misdiagnosis and suboptimal treatment with a negative outcome on the disease [2,3]. For instance, laser trabeculoplasty requires an open angle, and trabecular meshwork pigment status is informative for setting adequate power [4]. Moreover, minimally invasive glaucoma surgery (MIGS), performed standalone or in combination with cataract surgery to reduce the number of IOP-lowering drugs, requires detailed identification of angle structures for the correct placement of the device [5]. High-risk diabetic patients and eyes suffering from vaso-occlusive retinal disease may regularly need the anterior chamber angle (ACA) assessed for signs of neovascular glaucoma [6]. Additionally, eyes treated with procedures for reopening the angle such as iridotomy or iridoplasty should have the angle assessed over time because angles may continue to close [7]. For all these reasons, gonioscopy is an essential skill for diagnosis and treatment. Since the introduction of gonioscopy in clinical practice, technology has evolved, offering more advanced methods to inspect the ACA that partially overcome shortcomings of slit-lamp gonioscopy [8]. In particular, slit-lamp gonioscopy is often limited by poor documentation of findings, steep learning curve, eye contact, partial agreement between examiners, impractical photography of the observed finding and limited grading or measurements [9,10]. This review critically discusses the clinical value of two of the most promising technologies for document the ACA, namely optical coherence tomography (OCT) and digital gonioscopy.

## 2. Methods

We performed a search of electronic databases such as PubMed and Web of Science to identify studies on digital gonioscopy using the keywords: “goniophotography”, “GS-1”, “automatic gonioscopy”, “gonioscopy”, “anterior segment OCT”, “optical coherence tomography”, “anterior chamber angle”, “iridocorneal angle”. Additional sources were obtained from the reference lists of the included literature. We imposed no language or publication year restrictions. Medical Subject Headings (MeSH) searches were also performed. This article is based on previously conducted studies and does not involve any new studies of human or animal subjects performed by any authors.

### 2.1. Gonioscopy: The Reference Standard

The first limbus indentation able to identify the ACA was performed in 1907 by Trantas. However, except for rare cases of keratoconus, keratoglobus, or severe myopia, a direct view of the ACA was not possible due to the principle of total internal reflection [11]. Contact lenses and prisms have been introduced to overcome this problem. In 1914, Salzmann proposed the first gonioscopy lens, and in 1968, Goldmann enabled the visualization of angle structures through the gonioprism still used in our clinical practice. Based on the lens used to perform slit-lamp gonioscopy, a direct or indirect approach to the ACA is possible in daily practice. Koeppe’s lens allows a direct visualization of the angle. Light passes from the anterior chamber through the cornea and this contact lens, allowing a direct examination of the ACA. The one or three-mirrored Goldmann lens or the four-mirrored Zeiss lens permit an indirect view of the angle structures. In this technique, the light from the AC is reflected in a mirror, providing an inverted view of the ACA. Compared to the three-mirrored lenses, in which one of the three mirrors points toward the ACA and the others provide views of the anterior retina and Ora Serrata, in the four-mirrored, such as Zeiss, Volk G, Posner, and Susmann, all mirrored lenses point toward the ACA. Moreover, their relatively small and flat contact area allows a dynamic gonioscopy, which is still considered the reference standard.

Indentation gonioscopy through a gentle pressure placed on the cornea pushed the aqueous humor into the ACA, allowing to discriminate between an appositional (reversible) versus synechial (irreversible) angle closure. Indeed, in case of an iridotrabecular contact or apposition, the angle will open, and the angle structures become visible, whereas, in the presence of peripheral anterior synechiae (PAS), the angle will remain the same in the affected areas. Indentation gonioscopy is also extremely useful to figure out a plateau iris.

The anatomical landmarks of the ACA identified through the gonioscopy examination from posterior to anterior are: the iris root insertion, the ciliary body (CB), the scleral spur (SS), the trabecular meshwork (TM) and the Schwalbe’s line (SL). To provide a standardized description of the ACA, many classification systems have been established over time. For example, based on the visibility of the anatomical structures of the ACA, the Scheie system, based on angularity, the Shaffer system, based on the insertion of the iris, angularity, configuration, and pigmentation of the posterior TM, the Spaeth system [12].

Gonioscopy plays an essential role in recognizing glaucoma subtype and establishing proper medical or surgical treatment interventions [4,5,13]. In addition to its use in the classification of glaucoma, gonioscopy aids in diagnosing secondary causes of outflow obstruction such as pigment, new vessels, iris cysts, tumors, pseudoexfoliative material, angle-recession or foreign bodies in the ACA [1,3,6].

Both the experience of the examiner and the illumination condition could affect the gonioscopy interpretation. For example, too much inadvertent pressure on the cornea or excessive light exposure during the examination could overestimate the anterior chamber angle width. Comparison with other methods to inspect the ACA are summarized by Table 1.

### 2.2. Anterior Segment OCT

#### OCT as Screening Posterior Pole Disease

OCT was introduced as a non-invasive methodology to inspect the ACA in 1994. At that time, the system had an axial resolution of 10 μm and a lateral resolution of 22 μm; the image acquisition rate was slow, and because 0.8-mm wavelength light cannot penetrate the sclera ACA structures were poorly visualized [14]. Time-domain OCT using 1.3-mm wavelength were then developed allowing increased penetration through scattering angle structures so that ACA morphology could be visualized with more detail. Using the scleral spur (SS) as a landmark, it was then possible to quantify the ACA width using parameters previously introduced for the UBM including the angle opening distance (AOD) at 500 μm from the scleral spur, the angle recess area (ARA) and the trabeculo-iris space [15]. With OCT technology, precise measurements made it possible to measure dynamic changes in the ACA occurring with dark-light variations [16], quantify ACA morphology modifications after laser peripheral iridotomy [17,18] or phacoemusification [19,20]. While OCT offers non-contact and is less dependent on observer’s training than slit-lamp gonioscopy, it also presents several drawbacks. Firstly, correct angle classification and measurement necessitate precise landmark identification. In a study comparing gonioscopy and OCT for ACA assessment in glaucoma patients, SS was identified only in 56% and 50% of quadrants with a time-domain OCT and a spectral-domain OCT, respectively. Over time spectral-domain (SD) systems have an increased image acquisition speed and improved image resolution compared with time-domain systems, the shorter wavelength of 840 nm limits tissue penetration and imaging of the ACA [21]. To overcome these limitations, the use of the Schwalbe lines as a landmark has been proposed and validated by several studies. Moreover, swept-source OCT permit fast image acquisition rate with higher tissue penetration compared to spectral-domain systems and resulted in more reliable ACA imaging; Secondly, OCT imaging does not permit acquiring features of the ACA that are essential for glaucoma diagnosis and classifications. Intensity and patterns of ACA pigmentation may offer clues on secondary causes of glaucoma as pseudoexfoliation or pigment dispersion. In case of neovascular glaucoma, fine abnormal neovessels of the ACA may precede the synechial angle closure and slit-lamp examination and slit-lamp gonioscopy should not be replaced by OCT for follow-up high risk patients; Finally, moderate agreement exists in the detection of angle closure between slit-lamp gonioscopy and OCT. The OCT systems tend to overestimate angle closure compared to slit-lamp gonioscopy as the reference standard [8,22]. Beside these limitations, OCT may foster ACA assessment by permitting imaging of the trabecular meshwork and on the Sclemm’s canal and the band of extracanalicular limbal lamina with relevance to diagnosis and treatments [23]. Recently, features of angle dysgenesis evaluated with OCT in juvenile onset open-angle glaucoma were associated with poor selective laser trabeculoplasty success making this technology complementary to gonioscopy [24]. Moreover, microscope-integrated OCT are commercially available and provide valuable information before, during and at the end of anterior segment or posterior pole surgeries [25].

### 2.3. Digital Goniophotography

**Figure 1:**
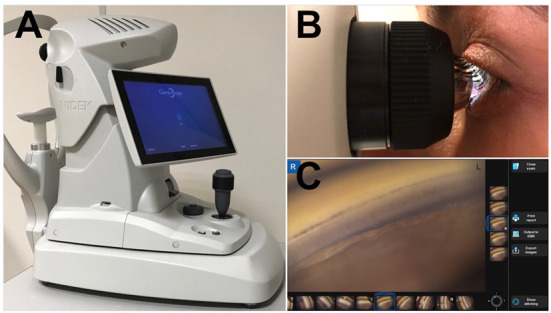
The GS-1 device. (**A**) GS-1 (Gonioscope GS-1; Nidek Technologies srl); (**B**) The examiner aligns the lens at the center of the cornea and then full 360° images of the angle are automatically acquired. Then lens is optically coupled with the cornea by a thin layer of gel; (**C**) The built-in software, controlled with multi-touch panel, permits acquisition and processing of the angle’s images.

A further advancement in ACA evaluation is represented by automatic gonioscopy that permits acquiring digital photographic documentation of the ACA. Figure 1 shows the NIDEK GS-1 Automated Gonioscope (NIDEK Co., Ltd., Gamagori, Japan) able to acquire 360° true color images of the ACA [26,27]. The instrument utilizes a multi-mirror prismatic lens with 16 reflecting facets, designed and developed specifically for this system and unique in its kind; in association with an illumination system provided with a white LED and a high-resolution color camera. The NIDEK multi-mirror prismatic lens was developed by optimizing each surface for automatic angle detection. Each of the 16 prism mirrored facets projects a white light onto a 22.5° portion of the irido-corneal angle. The camera takes 17 pictures at varying focal planes, from each of the 16 gonioprism facets in order to obtain a good level of focus. The best focused image acquired in every facet is automatically selected by the software and can be rendered in a unique, linear or circular, image of the entire irido-corneal angle called Stitching.

#### 2.3.1. Acquisition Protocol

The examination takes at least about 1 min, thus requiring good fixation and reasonable patient cooperation; it is advisable to perform it after instillation of topical anaesthetic. This system uses immersion gels to ensure patient comfort, the multi-mirror prism lens is not designed to contact directly the cornea.

The acquisition procedure is divided into two phases, the first involving the correct alignment of the camera with the ocular angular structures. The images from the upper, lower, nasal and temporal areas of the eye will appear on the instrument screen; the device indicates the state of alignment suggesting the movements to be made by the operator in order to get the best image quality. A first click on the acquisition joystick allows obtaining an automatic alignment of the angular structures, which the software is able to recognize autonomously regardless of the eyes color.

The second phase is the actual acquisition; once the correct viewing of the angle in at least three of four sectors has been achieved, the instrument automatically acquires the sixteen gonioscopic images at various focal planes by the rotating system. It is possible to make a manual acquisition by simply clicking the joystick again if the ideal alignment established by the software is not reached.

**Figure 2:**
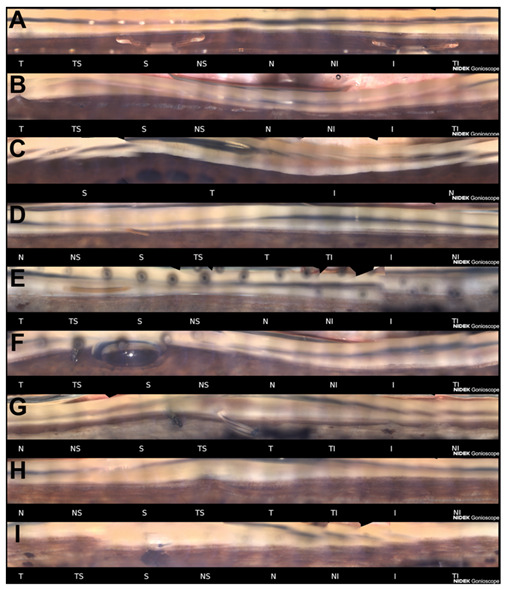
Examples of pathological and post-surgical findings imaged with GS-1. (**A**) Angle-supported phakic lens used in the past to correct myopia. Angle is open and moderately pigmented; (**B**) This eye was recently treated with phacoemulsification for angle closure. Previously, argon laser peripheral iridoplasty was performed. Some peripheral anterior synechiae are visible (T, N); (**C**) Angle closure. No angle anatomical structures are identifiable; (**D**) XEN gel stent implanted in a patient affected by exfoliative glaucoma (S). The ciliary band is visible for the majority of the angle. Sampaolesi line is well identifiable in the inferior quadrant; (**E**) XEN gel stent implanted after a failed ab externo canaloplasty (S). A 10-0 prolene suture in the Schlemm’s canal had been placed to maintain the canal under tension and therefore patent; (**F**) Ex-PRESS filtration device imaged few hours after the surgery (TS). A small air bubble is still present in the superior quadrant; (**G**) After a failed flap Ex-PRESS surgery, a Baerlveldt drainage device was implanted (TS). The tube is still closed by a prolene suture placed to prevent hypotony in the early postoperative period; (**H**) Deep sclerectomy with iris adhesion to the filtrating area (TS). Emulsified silicone oil is identifiable in the superior quadrant; (**I**) Iridectomy and the patent internal ostium of a trabeculectomy (S). Anterior chamber angle sectors: T = Temporal; TS = Temporal-Superior; S = Superior; NS = Nasal-Superior; N = Nasal; NI = Nasal-Inferior; I = Inferior; TI = Temporal-Inferior.

Each of the sixteen acquired sectors can be evaluated and saved by the operator with the intent of selecting the image captured with the best focus helped by the software that automatically highlights the most in-focus image for each sector. With all sixteen sections it is possible to create a single panoramic view called Stitching, linear or circular, which represents the entire structure of the iridocorneal angle. The 360-degree view of the angle is available in 3 available formats (16-section display, a circular display, or a linear display). Images can be saved in the built-in storage system or exported and transmitted for remote assessment. Figure 2 shows examples of pathological and post-surgical findings imaged with GS-1.

#### 2.3.2. Clinical Studies on GS-1

A pilot study on GS-1 performance reported the clinical utility of the instrument in assessing various clinical conditions including angle-closure, narrow angle and surgical devices and postsurgical conditions including CyPass, iStent, XEN, Baerveldt tube shunt, angle recession with iridodialysis, angle neovascularization, pigment dispersion, post-trabeculectomy sclerostomy and surgical iridectomy, and post-laser peripheral iridotomy [27]. Post-surgical findings were also evaluated by Barão et al. who analyzed the location of the XEN stent imaged by GS-1. They found that that different locations of XEN did not seem to significantly impact late success and that combined implantation of XEN with phacoemulsification does not seem to influence stent location [28]. More recently, Matsuo et al. used the GS-1 device to investigate the pattern of peripheral anterior synechia (PAS) formation after ab-interno trabeculotomy and they found that PAS formation rate was significantly higher within the incision compared to other angle sectors. GS-1 was also used to assess the pigmentation in the ACA using automatic and manual method with good agreement between the two techniques [29]. Agreement with slit-lamp gonioscopy has not yet been well characterized. In a pilot study, Texeira et al. reported that manual and automated gonioscopy showed only slight agreement for the assessment of iridocorneal angle opening status. In particular, angle closure was detected in 23.4% with slit-lamp gonioscopy in comparison with 4.3% using GS-1 [30]. Additionally, agreement between different graders of the ACA images acquired with the GS-1, although limited, could be improved by sufficient training and a solid consensus [29]. Peroni et al. recently better characterized the agreement on identification of the ACA structures between graders. Using a software annotation tool that permitted tracing the contours of anatomical layers, Peroni et al. found that scleral spur had the minimum agreement whereas it was best on iris root, trabecular meshwork and cornea [31]. Based on these studies, deep learning semantic segmentation algorithms for processing images acquired by GS-1 are currently in development [32,33,34].

### 2.4. Angle Evaluations in Virtual Clinics and Telemedicine

Poor accessibility to appropriate ophthalmological care is associated with inaccurate diagnosis and treatment of glaucoma. In particular, glaucoma requires prompt diagnosis and classification for an effective treatment as well as periodic follow-up visits to detect progression that requires additional therapies. Moreover, the number of patients affected by glaucoma is increasing worldwide and with the current COVID-19 pandemic healthcare systems are under pressure like never before [35,36]. Virtual glaucoma clinics are designed to acquire patient’s data in a reliable and efficient way by technicians to be reviewed by an ophthalmologist, thus maximising appointment capacity and reducing waiting times. There is evidence that patients report higher levels of satisfaction of this format and non-inferior knowledge of their condition than those seen in standard clinics [37]. Among the essential exams for glaucoma management there are slit-lamp examination, standard automated perimetry, optic nerve imaging or photography, tonometry, and ACA assessment. Among methods for angle assessments, both goniophotography and OCT have been tested for remote grading showing levels of agreement ranging from fair to excellent with the ground truth gonioscopic examination [38,39]. When considering ACA assessment without standard slit-lamp gonioscopy, OCT could have advantages over goniophotography for screening for angle closure disease because it does not require contact and is easier to perform. However, goniophotography is more informative for detecting signs of secondary glaucoma or inspecting angle sectors subject to previous angle surgeries or device placement. EyeCam permits acquiring clinically gradable images but it is limited by an acquisition time of approximately 5 to 10 min per eye and by its requirement for supine patient positioning [39]. Viceversa, GS-1 is performed with the patient in sitting position and the entire angle is photographed in less than a minute, probably making this technology more appealing for a virtual clinics setting.

## 3. Discussion

Technologies for the assessment of the ACA have evolved in recent years thanks to advancements in the miniaturization of mechanical, optical, and electronic devices. Also, software development and image processing have permitted the realization of precise measurements of the morphology of the ACA [40]. On the other hand, slit-lamp gonioscopy is still considered the reference standard and an essential skill for the ophthalmologist [1]. New technologies have made it possible to acquire digital images of the ACA, ameliorating documentation, comparisons over time and permitting remote diagnosis and image analysis. Both OCT and digital gonioscopy are fast and easy techniques to perform and could be suitable for virtual clinics, whereas slit-lamp gonioscopy requires considerable training. Actual therapeutical algorithms and angle classification are based on slit-lamp gonioscopy findings and there is uncertainty over whether the use of new methods to assess the ACA are associated with better clinical outcomes. Digital gonioscopy and OCT role in patient’s management is not yet completely understood. Ultrasound biomicroscopy, even if not discussed in detail in this review, has a role when complex angle-closure mechanisms are suspected because of its ability to image behind the iris [41]. All the complementary techniques for imaging the ACA should be better considered as complementary exams to slit-lamp gonioscopy and not as alternatives [41]. OCT has shown a potential role in predicting the outcome of laser treatments [42,43,44,45]. Digital gonioscopy has been shown useful in documenting post-operative and abnormal findings of the ACA and has a great potential for teaching gonioscopy. Deep learning algorithms for automatic classification of the angle are on the horizon and their validity in the clinical setting should be accurately evaluated [31,32,33].

## Figures and Tables

**Table 1:** Advantages and disadvantages of three different methods to inspect the anterior chamber angle.

	Slit-Lamp Gonioscopy	Digital Goniophotography	OCT *
Advantages	Reference standard Evaluation of angle characteristics (e.g., width, iris insertion, iris profile, pigmentation, vessels) Essential for lasers Compression gonioscopy (discrimination between synechial or appositional closure)	Documentation Real color photo of the anterior chamber angle Angle characteristics (e.g., iris insertion, pigmentation, vessels) Fast 360° angle acquisition Easy to perform Suitable for recording pre and post-op findings (e.g., angle devices) Suitable for deep-learning and automatic classification (not commercially available)	Widely available technology Easy to perform Built-in software to perform angle measurements Real “dark room exam” Suitable for deep-learning and automatic classification (not commercially available)
Disadvantages	Poor documentation Steep learning curve Not possible to visualize structures behind the iris No measurements Photography with a photographic slit-lamp is time consuming	No indentation Iris profile not evaluable Partial agreement with slit-lamp gonioscopy Not possible to visualize structures behind the iris	Landmarks sometimes hard to recognize Partial agreement with slit-lamp gonioscopy Lacking information on essential findings (e.g., pigments, neovessels) Not possible to visualize structures behind the iris

* Many devices and technologies exist and performance varies greatly between instruments. Spectral domain has poor tissue penetration compared with time-domain and swept-source technology.
